# Facile Synthesis and Origin of Enhanced Electrochemical Oxygen Evolution Reaction Performance of 2H-Hexagonal Ba_2_CoMnO_6−δ_ as a New Member in Double Perovskite Oxides

**DOI:** 10.1021/acsomega.2c05627

**Published:** 2022-11-21

**Authors:** Tuncay Erdil, Ersu Lokcu, Ilker Yildiz, Can Okuyucu, Yunus Eren Kalay, Cigdem Toparli

**Affiliations:** † Department of Metallurgical and Materials Engineering, Middle East Technical University, 06800 Ankara, Turkey; ‡ Department of Metallurgical and Materials Engineering, Eskisehir Osmangazi University, 26040 Eskisehir, Turkey; § Central Laboratory, Middle East Technical University, 06800 Ankara, Turkey

## Abstract

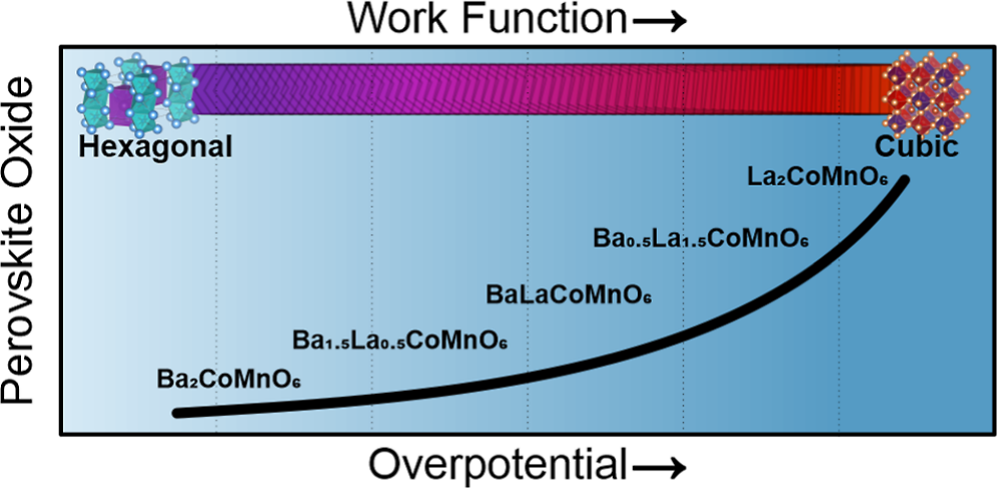

Perovskite oxides have been considered promising oxygen evolution reaction (OER) electrocatalysts due to their high intrinsic activity. Yet, their poor long-term electrochemical and structural stability is still controversial. In this work, we apply an A-site management strategy to tune the activity and stability of a new hexagonal double perovskite oxide. We synthesized the previously inaccessible 2H-Ba_2_CoMnO_6−δ_ (BCM) perovskite oxide via the universal sol–gel method followed by a novel air-quench method. The new 2H-BCM perovskite oxide exhibits outstanding OER activity with an overpotential of 288 mV at 10 mA cm^–2^ and excellent long-term stability without segregation or structural change. To understand the origin of outstanding OER performance of BCM, we substitute divalent Ba with trivalent La at the A-site and investigate crystal and electronic structure change. Fermi level and valence band analysis presents a decline in the work function with the Ba amount, suggesting a structure–oxygen vacancy–work function–activity relationship for Ba_*x*_La_2–*x*_CoMnO_6−δ_ (*x* = 0, 0.5, 1, 1.5, 2) electrocatalysts. Our work suggests a novel production strategy to explore the single-phase new structures and develop enhanced OER catalysts.

## 1 Introduction

Electrochemical oxygen evolution reaction (OER) is a central reaction for various energy devices such as water electrolyzers, fuel cells, or rechargeable metal–air batteries.^1−6^ Yet, the sluggish kinetics of OER cause high anodic overpotential, lowering the overall efficiency of these devices.^7−14^ At present, platinum group elements (IrO_2_, RuO_2_, and Pt/C) are widely applied to mitigate the anodic overpotential.^15−17^ However, they are costly and scarce and exhibit poor long-term stability; thus, their large-scale utilization is not feasible.

In the search for economically viable and robust OER electrocatalysts, perovskite oxides have emerged rather than metal-based/metal oxide catalysts^18−23^ due to their adjustable A and B sites and, thus, physicochemical properties. Typically, the perovskite oxide (ABO_3_) structure involves 3d transition metals at the B-site, and these metals are active for OER.^24−27^ Versatile crystal and electronic structures can be achieved via altering the A-site and/or B-site elements in a perovskite oxide structure. Hence, the perovskite oxide structure offers a great platform for establishing the material’s nature and electrochemical performance relationship.^28−30^ Several times, it has been reported that the OER activity of perovskite oxides is closely interrelated with the electronic structure of B-site metal, lattice oxygen participation, and oxygen vacancies.^31−36^ In fact, this implies that any strategy manipulating the B-site element can result in a variation in the OER activity. Although in most studies, oxidation of the B-site element has been varied via substitution of a B-site element,^37−41^ A-site management strategy-induced OER performance has been less explored. Substitution of a trivalent ion with a divalent ion at the A-site can lead to rearrangement of the charge balance of the structure and vacancy formation.^27,32,42−45^ For example, the substitution of divalent Sr at the A-site of LaCoO_3_ was shown to increase electrical conductivity and thus enhance OER performance.^27^ Sr doping into La_1–*x*_Sr_*x*_CoO_3−δ_ has changed the OER mechanism from the adsorbate evolution mechanism to the lattice-oxygen-mediated mechanism.^46^ However, it is widely reported that Sr substitution at the A-site can lead to segregation and surface reconstruction during the electrochemical reaction.^47−49^ In addition to this, Ba_0.5_Sr _0.5_Co_0.8_Fe_0.2_O_3−δ_,^50^ a well-known catalyst, also suffers from crucial structural instability under OER conditions and transforms to an amorphous state. Among the possible divalent ion choices for the A-site, Ba is an interesting element since it forms the hexagonal structure due to its ionic radius, while Sr, for example, constitutes a cubic structure. The key difference between the cubic and hexagonal structures is that B cations are only connected to corner-sharing points in the cubic structure, while in the hexagonal structure they are connected to face-sharing octahedral sites. Theoretical calculations showed that face-sharing octahedral sites play a vital role in the high OER activity of hexagonal perovskite oxides over cubic ones.^51−54^ Moreover, the ionic size of Ba is greater than that of Sr^2+^ and La^3+^, and it has been reported that increasing the size of the A-site cation would increase the O–M bond angle leading to an increase in the electrical conductivity.^55^

Inspired by the above-mentioned discussions, we synthesized a previously inaccessible new 2H-Ba_2_CoMnO_6−δ_ (BCM) double perovskite oxide via a novel air-quenching method. 2H-BCM achieves a current density of 10 mA cm^–2^ at an overpotential of 288 mV. Furthermore, 2H-BCM exhibits an outstanding stability of ∼60 h in a 0.1 M KOH electrolyte. Structural analysis through high-resolution transmission electron microscopy (HRTEM) after ∼60 h of the OER stability test shows no structural change or amorphization. In order to understand the outstanding OER performance of 2H-BCM, we investigated La substitution at the A-site to reach a cubic phase. The experimentally measured work function shows conductivity and the oxidation state of cations, and the oxygen vacancy concentration decreases with La substitution. The results of both structural and electrochemical data show that divalent Ba substitution at the A-site is a successful strategy to obtain enhanced OER activity and stability.

## 2 Experimental Section

### 2.1 Synthesis of Perovskite Oxides

Ba_*x*_La_2–*x*_CoMnO_6−δ_ (*x* = 0, 0.5, 1, 1.5, 2) powders were synthesized by a modified sol–gel Pechini method. A stoichiometric amount of Ba(NO_3_)_2_, La(NO_3_)_3_·6H_2_O, Co(NO_3_)_2_·6H_2_O, and Mn(NO_3_)_2_·4H_2_O was dissolved in deionized water (18.2 MΩ·cm). Following this, a metal cation, acrylamide (AC), and citric acid (CA) were mixed into the solution as complexing agents with a molar ratio of 1:9:3. The solution was stirred on a hot plate at 100 °C till a gel was formed. After evaporation of water, the gel was dried in an oven at 200 °C for ∼10 h, followed by calcination at 600 °C for 15 h. Powders were annealed and air-quenched at 1300 °C to reach a single-phase double perovskite oxide.

### 2.2 Material Characterization

The crystal structures of the series of Ba_*x*_La_2–*x*_CoMnO_6−δ_ (*x* = 0, 0.5, 1, 1.5, 2) were studied by powder X-ray diffraction (XRD, Rigaku) with Cu Kα radiation (*l* = 1.5406 Å) in a 2θ range of 10–90°. The refinement of the XRD patterns was conducted with the Rietveld refinement method using the EXPGUI interface and GSAS program. The morphology and microstructure of the samples were characterized using a field-emission high-resolution transmission electron microscope (Tecnai G2 F30) and a high-resolution field-emission scanning electron microscope (FEI Nova NanoSEM 430). High-resolution transmission electron microscopy (HRTEM) was used to obtain high-resolution and high-angle annular dark-field (HAADF) micrographs and corresponding energy-dispersive spectroscopy (EDS) element mapping and also selected area electron diffraction (SAED) patterns. The Brunauer–Emmett–Teller (BET) method within the relative pressure range *P*/*P*_0_ = 0.06–0.30 was used to calculate the specific areas. The chemical composition, nature of the perovskite oxides, and work function measurements were studied using X-ray photoelectron spectroscopy (XPS, PHI 5000 Versa Probe spectrometer) with Al Kα radiation. All the peaks were calibrated with a standard C 1s spectrum at 284.6 eV. For work function measurements, a previous approach was applied.^56^

### 2.3 Electrochemical Characterization

The electrochemical measurements were performed on a three-electrode system using a rotating glassy carbon (GC) disk electrode (RDE, BASI) with a GAMRY Reference 3000 potentiostat/galvanostat/ZRA. A Ag/AgCl electrode was used as a reference, and a platinum wire was used as the counter electrode. All tests were measured in an O_2_-saturated solution of 0.1 M KOH prepared from deionized water (18.2 MΩ) and KOH pellets (Alfa, 99.99%). All potentials versus Ag/AgCl were normalized to the reversible hydrogen electrode (RHE) according to the Nernst equation, *E*_vs RHE_ = *E*_vs Ag/AgCl_ + 0.059 × pH + 0.1976pH for 0.1 M KOH = 12.6, and *iR*-corrected to compensate for solution resistance. To prepare the working electrode, 8 mg of the perovskite oxide, 5 mg of Super-P carbon, and 50 μL of Nafion solution (5 wt %, Sigma-Aldrich) were dispersed in 2 mL of ethanol. The mixture was ultrasonicated for ∼3 h to obtain homogeneous ink. Linear sweep voltammetry (LSV) measurements were performed in the range of 0.2–1.1 V versus Ag/AgCl at a scan rate of 10 mV s^–1^. The mass activity (MA) and specific activity (SA) are calculated according to the equations given: MA = *J*/*m* and SA = *J*/(10 × *m* × SBET), where *J*, *m*, and SBET are the current density (mA cm^–2^), the mass loading (0.557 mg cm^–2^), and the BET surface area (m^2^ g^–1^), respectively. In order to realize the Tafel analysis under steady-state conditions, Tafel analysis was performed through the chronoamperometry (CA) method applied in a potential range of 0.4–0.59 V versus Ag/AgCl at a 0.01 V increment. Electrochemical impedance spectroscopy (EIS) was performed using an AC voltage with 10 mV amplitude within the frequency range of 1 × 10^5^ to 1 × 10^–2^ Hz and recorded at 1.641 V versus the RHE. EIS is performed the same as LSV (0.1 M KOH), and the RDE setup is used. To investigate the long-term durability, the chronopotentiometry (CP) test was performed at a constant current to maintain an initial current density of 10 mA cm^–2^ for ∼60 h. Mott–Schottky (MS) analysis was conducted with EIS at different applied potentials from open circuit voltage (OCV) −0.5 to 0.6 V versus Ag/AgCl in 50 mV increments. The space charge capacitance is calculated using the equation *C* = −1/2π*vZ*″. *Z*″ is the imaginary part of the impedance at the constant frequency *v* = 10 Hz.

## 3 Results and Discussion

### 3.1 Structure of the Catalysts

**Figure 1:**
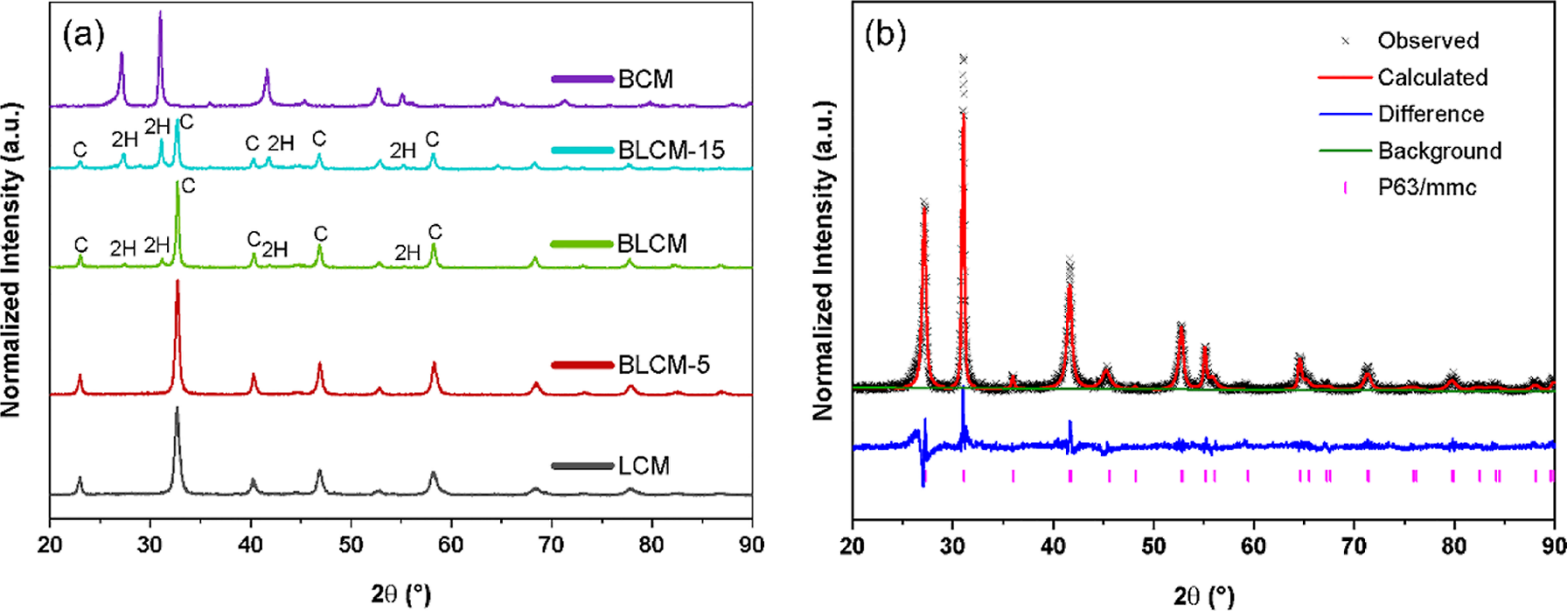
(a) XRD patterns of the double perovskite series of BCM, Ba_1.5_La_0.5_CoMnO_6−δ_ (BLCM-5), BaLaCoMnO_6−δ_ (BLCM), Ba_0.5_La_1.5_CoMnO_6−δ_ (BLCM-15), and La_2_CoMnO_6−δ_ (LCM). (b) Rietveld refinement profile of XRD for BCM.

In order to reach a single-phase composition, we fabricated several Ba_2_Co_*x*_Mn_2–*x*_O_6−δ_ samples with different Co/Mn ratios and annealed them at different temperatures. For the simplicity of visualization, only the data from single phases are shown in Figure 1 (see Figure S1). Based on XRD patterns in Figure S1a, annealing at 1100 °C is not enough to obtain a pure double perovskite structure; single perovskite phases (BaCoO_3_, BaMnO_3_) and side phases were also observed in the XRD pattern. When the samples were annealed at 1300 °C and rapidly air-quenched, the major diffraction peaks of the 2H-BaMnO_3_-type structure were observed, and the peaks of side phases disappeared, especially for the BCM sample. Ba_2_Co_0.5_Mn_1.5_O_6−δ_ still contains minor secondary phases. The Ba_2_Co_1.5_Mn_0.5_O_6−δ_ composition was not synthesized successfully due to partial melting at 1300 °C (see Figure S1). Co-rich inter-oxidic phases may trigger this partial melting. In Figure 1b, Rietveld refinement analysis of annealed and air-quenched BCM at 1300 °C indicates that the crystal structure (Pearson’s Crystal Data: 1900378) is 2H-hexagonal with the space group *P*6_3_/*mmc* (Figure 1b) and lattice constants *a* = 5.77 Å, *c* = 4.37 Å. Furthermore, Figure 1a shows XRD patterns of the series of Ba_*x*_La_2–*x*_CoMnO_6_ (*x* = 0, 0.5, 1, 1.5, 2). Table S1 summarizes the crystal structures, lattice parameters found from Rietveld refinement analysis, and the Goldschmidt tolerance factor of Ba_*x*_La_2–*x*_CoMnO_6−δ_ (*x* = 0, 0.5, 1, 1.5, 2). According to the XRD pattern, La_2_CoMnO_6−δ_ has an ideal cubic perovskite structure with an *Fm*3̅*m* space group. Typically, the crystal structure of the perovskite oxides is related to the Goldschmidt tolerance factor, *t*_f_ = (*r*_A_ + *r*_O_)/[√2(*r*_B_ + *r*_O_)], where *t*_f_ is the tolerance factor and *r*_A_, *r*_B_, and *r*_O_ are the average radii of A-site cations, B-site cations, and oxygen anions, respectively. Accordingly, *t*_f_ values between 0.8 and 1.0 represent the formation of the cubic structure, while a *t*_f_ value greater than 1.0 would result in a hexagonal structure formation. Thus, the XRD results are well matched with the Goldschmidt tolerance factor.

**Figure 2:**
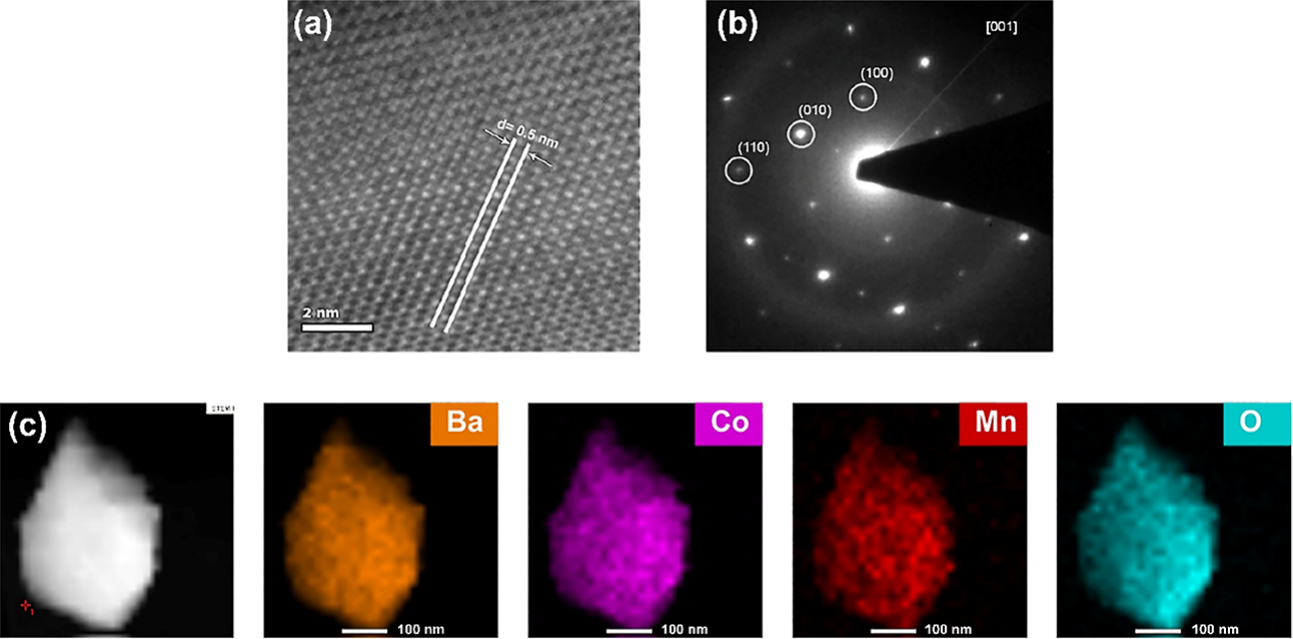
(a) HRTEM image of BCM, (b) SAED patterns along the [001] axis for BCM, and (c) HAADF image and the corresponding EDS element mappings of Ba, Co, Mn, and O in BCM.

To further investigate the 2H hexagonal crystal structure of BCM, HRTEM and corresponding SAED techniques were performed. Figure 2a shows the HRTEM image of hexagonal BCM, and it can be seen that one atom is placed at the center, and six atoms are around it. Figure 2b shows the corresponding SAED patterns of BCM. The pattern reveals a hexagonal feature of BCM matching well with the results of XRD and Rietveld refinement. These results complement the formation of the 2H layered hexagonal perovskite oxide structure. EDX mapping (Figure 2c) was conducted to analyze the distribution of the elements in the BCM double perovskite. The results showed that the Ba, Co, Mn, and O elements are homogeneously distributed in the structure. Moreover, elements of other synthesized double perovskites are also homogenously distributed (Figure S3).

### 3.2 Electronic Structure of the Catalysts

**Figure 3:**
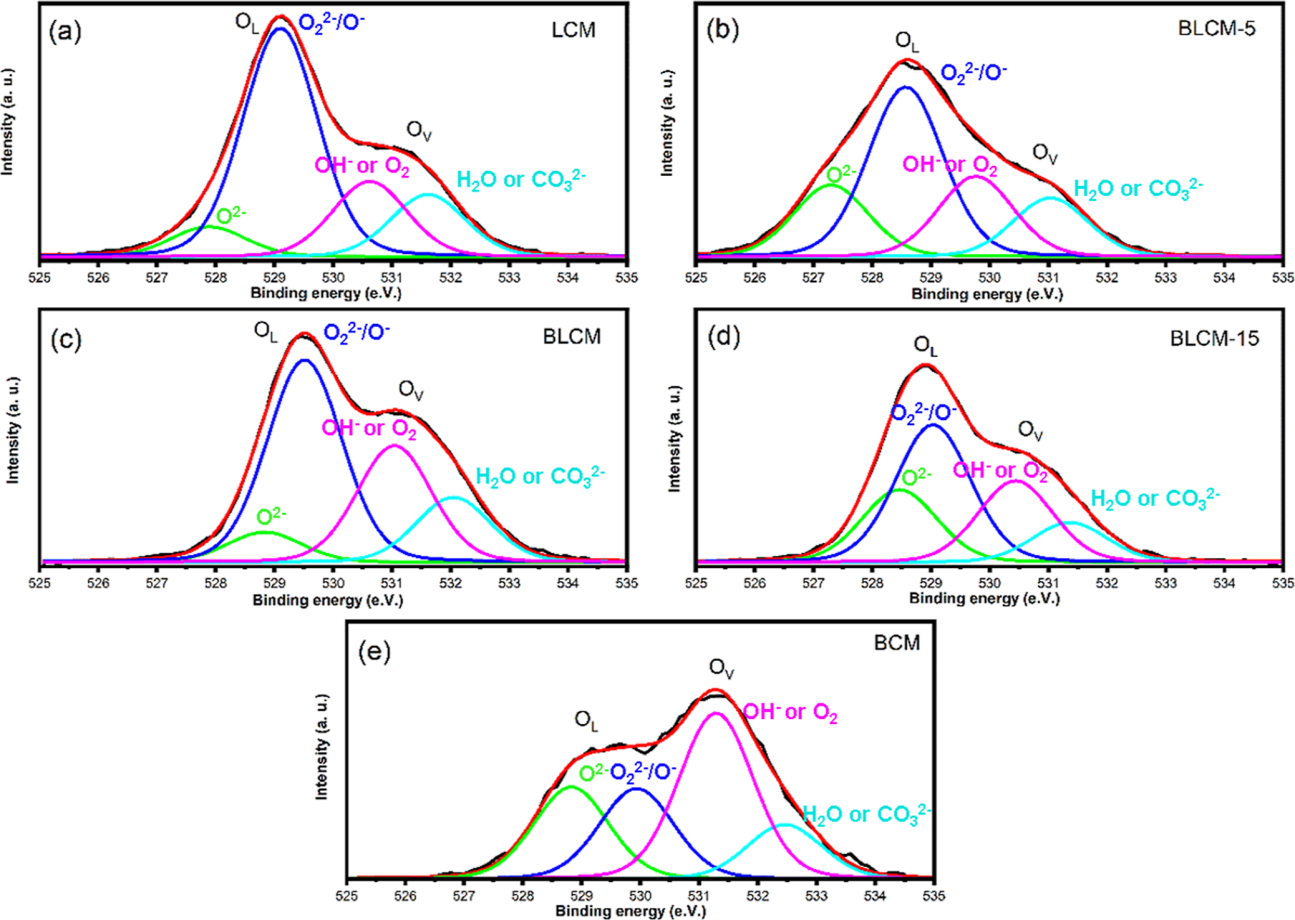
XPS core-level spectra of (a) LCM, (b) BLCM-5, (c) BLCM, (d) BLCM-15, and (e) BCM.

Substitution of the trivalent ion with divalent ion would result in an imbalance in the net charge, and thus, it must be compensated to maintain the overall electrical neutrality of the perovskite oxide structure. In order to keep the charge balance, there can be either increase in the oxidation state of B-site cations or the formation of additional oxygen vacancies. Therefore, to probe the changes in the electronic structure due to Ba substitution, XPS analysis was performed. Survey spectra of the series of Ba_*x*_La_2–*x*_CoMnO_6−δ_ (*x* = 0, 0.5, 1, 1.5, 2) show no impurity elements present in the structure (see Figure S4). Figure S5a presents XPS core-level spectra of Co 2p. Co 2p spectra shown in Figure S5a have a strong satellite of Co^2+^, emphasizing that Co^2+^ is dominant in the cubic LCM structure.^57^ As the Ba content increases, the intensity of the satellite from Co^2+^ decreases, and the weak satellite from Co_3_O_4_ appears together with hexagonal phase formation. Mn 2p core-level spectra of the catalysts are shown in Figure S5b. The satellite at 646 eV is an indication of the oxidation state of Mn^2+^.^9,58^ As the Ba amount increases in the structure, these weak satellites disappear, and the Mn 2p_3/2_ peak becomes narrower, suggesting Mn_2_O_3_ presence. The O 1s spectra are shown in Figure 3. The fitted peaks at around 529.5 and 531.2 are associated with lattice oxygen and oxygen, respectively.^57^ The peak related to the oxygen vacancy formation at ∼531 eV becomes more dominant in the spectra of BCM.

### 3.3 Electrochemical OER Activity of the Catalysts

**Figure 4:**
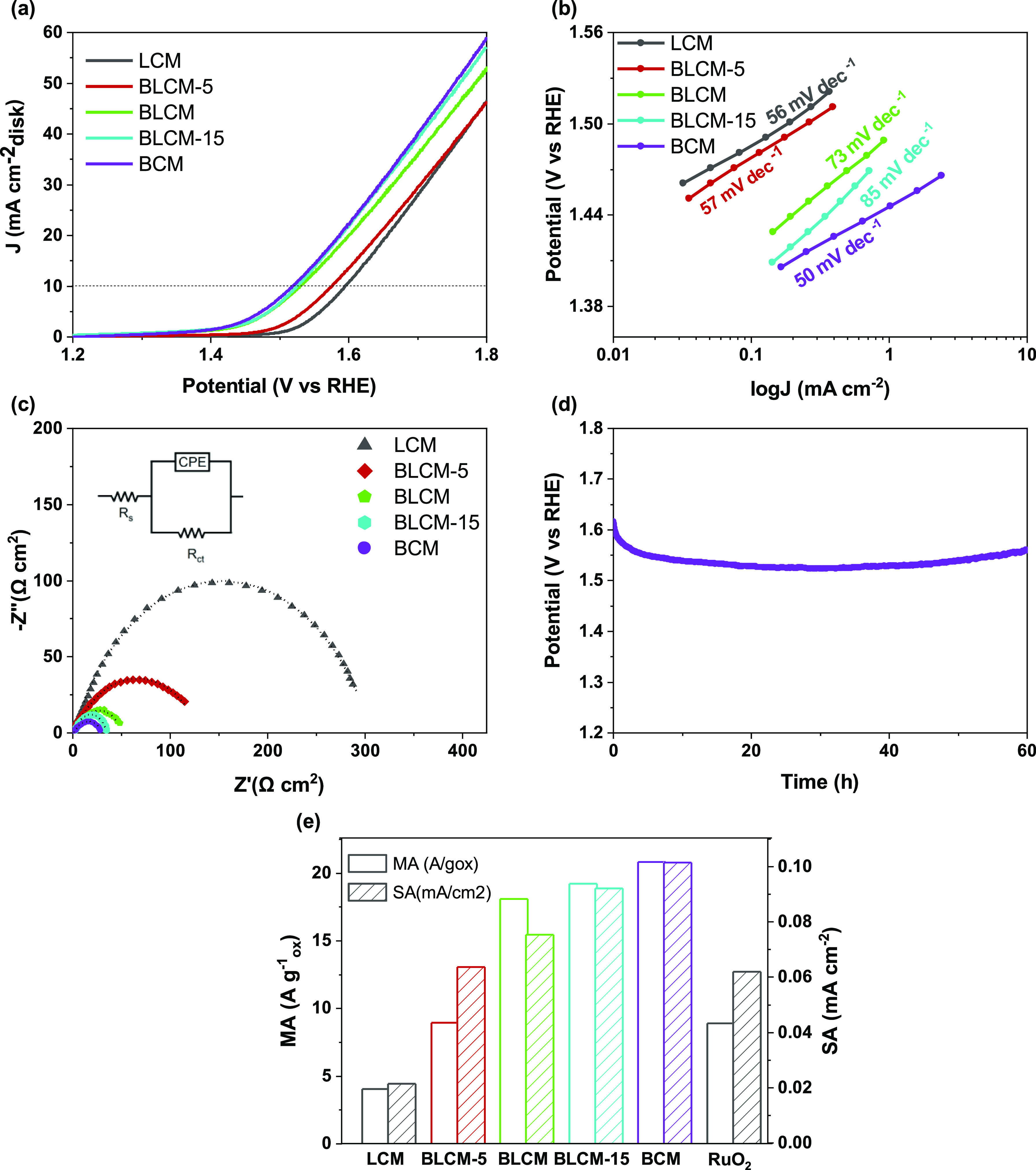
(a) OER activity curves of double perovskite electrocatalysts, (b) Tafel plots obtained from steady-state measurements, (c) CP stability curve of BCM at a current density of 10 mA cm^–2^, (d) EIS of catalysts at a potential of 1.641 V vs RHE, and (e) SA and MA of LCM, BLCM-5, BLCM, BLCM-15, BCM, and RuO_2_.^51^ RuO_2_ data reproduced from Luo, Q.; Lin, D.; Zhan, W.; Zhang, W.; Tang, L.; Luo, J.; Gao, Z.; Jiang, P.; Wang, M.; Hao, L.; Tang, K. Hexagonal Perovskite Ba_0.9_Sr _0.1_Co_0.8_Fe_0.1_Ir_0.1_O_3−δ_ as an Efficient Electrocatalyst toward the OER. *ACS Appl. Energy Mater.***2020,***3 (7),* 7149–7158. Copyright 2020 American Chemical Society.

The OER activity of the double perovskite oxide series Ba_*x*_La_2–*x*_CoMnO_6−δ_ (*x* = 0, 0.5, 1, 1.5, 2) was measured using a standard three-electrode system in O_2_-saturated 0.1 M KOH using the RDE as the working electrode. The IR-corrected LSV curves normalized by the geometric area of the GC electrode (0.07068 cm^2^) are shown in Figure 4a. The OER catalytic activity of the double perovskite series of Ba_*x*_La_2–*x*_CoMnO_6−δ_ (*x* = 0, 0.5, 1, 1.5, 2) increases upon increasing the Ba doping level (*x*). The order of OER activity is BCM > BCM-15 > BLCM > BLCM-5 > LCM. LCM and BLCM-5 are pure cubic phases, while BLCM, BLCM-15, and BCM include the hexagonal phase. The overpotential of the catalysts including the hexagonal phase, BLCM (η = 300 mV), BCM-15 (η = 295 mV), and BCM (η = 288 mV), is significantly lower than that of pure cubic ones BLCM-5 (η = 346 mv) and LCM (η = 365 mV). The performance of the catalyst in higher KOH concentrations (Figure S10), 1 and 6 M, is also measured, and exactly the same catalytic activity trend is observed. Tafel slopes were obtained by collecting steady-state currents via multistep CA. Multistep CA was performed in a potential range of 0.4–0.59 V versus Ag/AgCl at a 0.01 V increment. The steady-state current after each potential is measured and converted to the current density by dividing the working electrode area. The multistep CA results are shown in Figure S5. The calculated Tafel slopes from CA experiments are 50, 85, 73, 57, and 56 mV dec^–1^ for BCM, BLCM-15, BLCM, BLCM-5, and LCM, respectively. Here, BCM shows the highest activity and favors the OER kinetics. EIS was performed to investigate the charge-transfer ability of the catalysts. The equivalent circuit fit by EIS data includes a solution resistance (*R*_s_), a constant-phase element, and a charge transfer resistance (*R*_ct_), as shown in Figure 4c. According to the model, the charge transfer resistance of BCM is 30.86 Ω which is smaller than that of the other catalyst tested in this work, 306, 131, 51, and 33 for LCM, BLCM-5, BLCM, and BLCM-15, respectively. This also implied that the intrinsic electronic conductivity of BCM is higher than that of the others. Moreover, the hexagonal crystal structure included perovskites, for example, BCM, BLCM, and BLCM-15, which have significantly smaller semicircle diameters, hence small resistance, compared with that of the cubic perovskites BLCM-5 and LCM. The CP method was applied to evaluate the catalytic durability of BCM^59^ at a constant current density of 10 mA cm^–2^, as shown in Figure 4d. BCM shows ∼60 h stability suggesting superior stability together with excellent activity than current state-of-the-art catalysts.

Perovskite oxide catalysts generally have low MA and SA due to high annealing temperatures applied during the synthesis procedure (e.g., below 1.6 V). Therefore, it is extremely important to understand the influence of the surface area and mass loading on the activity to evaluate the intrinsic activity of the perovskite oxide-based electrocatalysts. The specific surface area of all particles was measured via N_2_ adsorption/desorption isotherm curves and calculated by the BET method (see Table S2). The specific surface area of the catalysts is more or less the same; thus, the effect of surface area on the electrocatalytic activity can be eliminated, and the effect of elements and doping can be investigated. The MA was also calculated to investigate the activity related to mass loading. Here, the mass loading of the catalyst is used to normalize MA. As shown in Figure 4e, there is a sharp increase in both MA and SA as the Ba amount increases in the structure. The current density per surface area of the catalyst is used to describe the SA. It is a close approximation to the turnover frequency (TOF), which is the volume of electrons moved through an active site each second. In order to research the intrinsic chemistry of electrocatalysts, SA is being employed widely. The TOF is equivalent to intrinsic activity, but the number of active sites is frequently unknown. The TOF values are well related to the SA graph, and the values and comparison with the literature are given in Figure S7 and Table S3.

**Figure 5:**
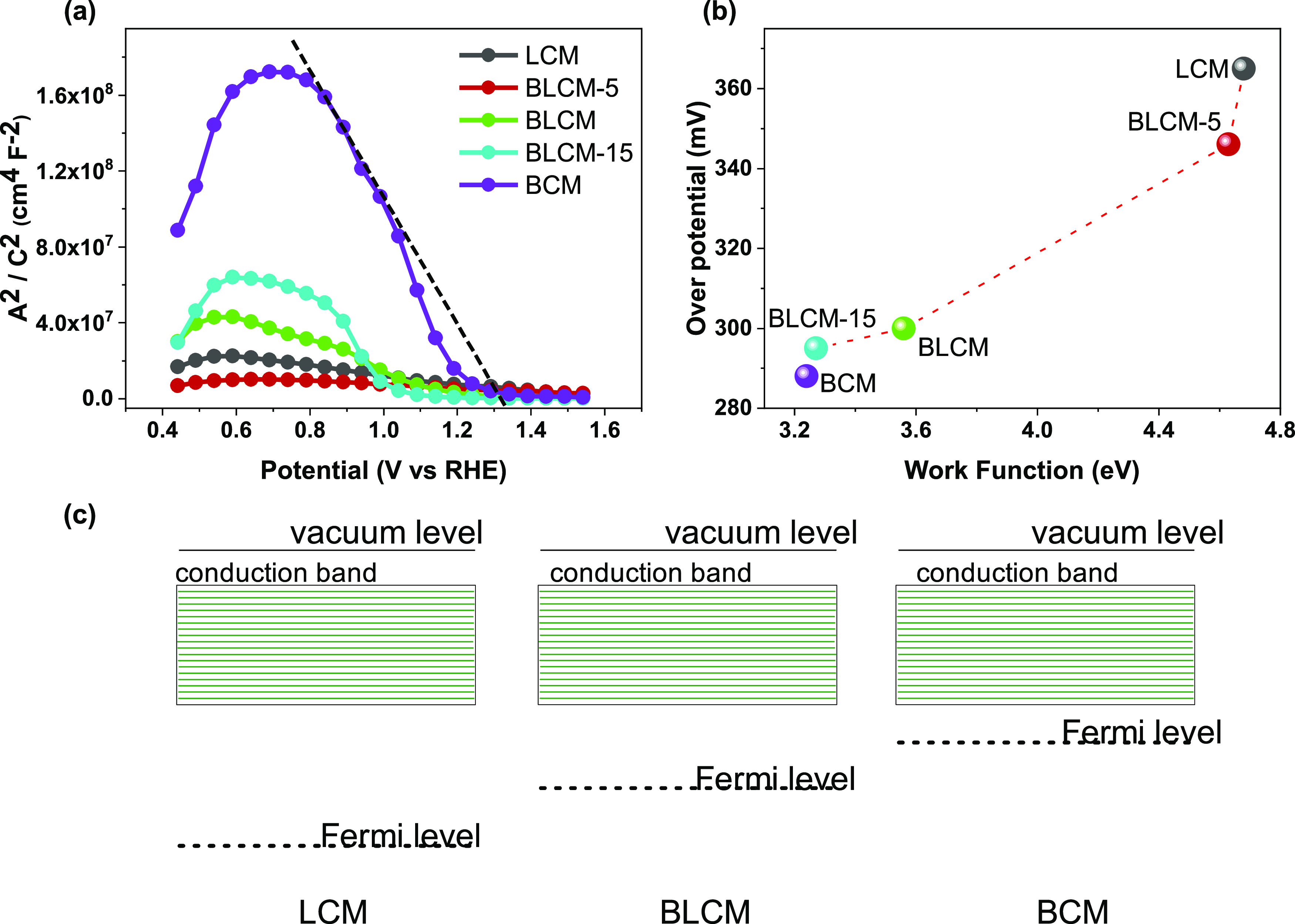
(a) MS plots of the double perovskite oxide electrocatalyst in alkaline medium, (b) correlation between the work function and overpotential, and (c) representative scheme of Fermi level alignment of LCM, BLCM, and BCM relative to vacuum.

To understand the origin of the enhanced electrochemical OER activity of BCM and realize the OER descriptor, we study the band bending behavior at the solid–liquid interface and the surface work function value of all catalysts. MS analysis was applied to investigate band bending behavior at the OER potential window. As shown in Figure 5a, all samples show n-type behavior (positive slope) from the OCV to approximately 0.8 V versus RHE. This indicates that due to downward band bending, the surface of the nominally p-type catalyst layers is in charge inversion. The transition between n-type and p-type (negative slope) behavior is observed around the 0.8 V versus RHE; the transition changes inversion to the hole depletion region.^33^ The maxima of the MS plots indicate the exact transition potential. The flat-band potential decreases from LCM to BCM. *E*_fb_ is measured from linear extrapolation of the MS plots; it yields 1.34 V versus RHE for BCM and over 1.86 V versus RHE for BLCM-5. The higher flat-band potential represents a strong limitation for the OER activity.^10,33^ Over *E*_fb_, the MS plots become flat; this indicates the presence of hole accumulation in the OER regime. Overall, all samples show hole accumulation in the OER potential region, and thus, it is unlikely that electronic charge transfer at the interface dominates the OER activity of LCM, BLCM-5, BLCM, BLCM-15, and BCM.

We then turn our interest to the surface work function of the catalyst since the work function of a material is considered to have a significant role in OER electrochemistry. Here, in this work, we experimentally investigated the work function of Ba_*x*_La_2–*x*_CoMnO_6−δ_ (*x* = 0, 0.5, 1, 1.5, 2) to find out a possible relation between the electrochemical OER activities (Figure 5b). To do so, a previously reported approach was applied to measure the work function via XPS Fermi level and valence band spectral analysis (see Figure S7 and Table S3). Here, it is important to highlight that the measured work function values should be considered a qualitative trend rather than a quantitative manner. The measured work function values decrease with Ba substitution, which is also compatible with EIS results, implying that the intrinsic conductivity of BCM is higher in the series. Modulation of surface electron affinity can be explained by elevating the electronegativity of Co and oxygen vacancy formation, which is in accordance with the Gordy–Thomas relation.^60^ In addition, according to previous reports and density functional theory calculations, the bond distance between B-site elements decreases, and the B–O bond distance increases in face-sharing octahedra, which may indicate that the B–B bond is metallic in character, and the material may have intrinsic metallic conductivity related to this bonding character.^51^ Here, considering that BCM has a 2H-hexagonal structure with fully face-sharing octahedra, this structural behavior may contribute to metallic character based on the above-mentioned discussion and yield a decrease in the work function due to enhancement in the electronic conductivity.

**Figure 6:**
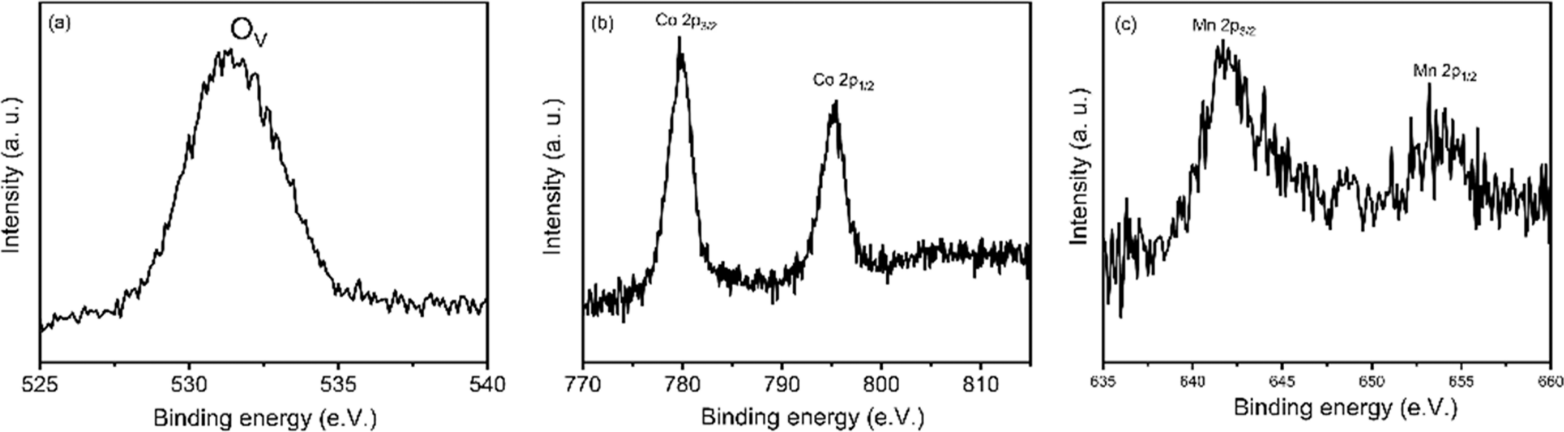
XPS core-level spectra of BCM after ∼60 h of the CA test (a) O 1s, (b) Co 2p, and (c) Mn 2p.

In general, long-term electrochemical and structural stability is a trade-off for OER electrocatalyst design. Thus, we investigated the nature of BCM after ∼60 h of the OER stability test. XPS analysis indicates no change in the chemical state of Co and Mn as shown in Figure 6b,c, while the concentration of oxygen vacancies in the structure increases, Figure 6a. These vacancies can easily participate in the reaction and can be a source of O_2_ production by contributing to charge transport.

**Figure 7:**
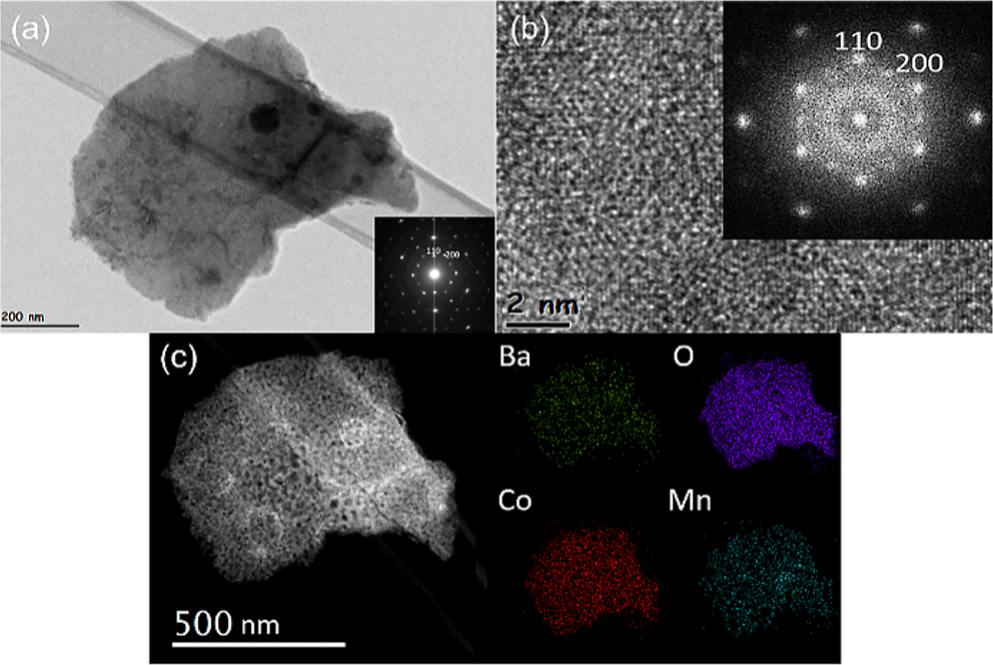
After ∼60 h of the CA test on BCM. (a) Bright-field image and SAED pattern (inside) along the [100] axis, (b) HR-TEM image and corresponding FFT pattern (inside) along the [100] axis, and (c) HAADF image and corresponding EDS element mapping of Ba, Co, O, and Mn.

Figure 7 shows the TEM images of hexagonal BCM after 60 h of the stability test. The SAED and fast Fourier transform (FFT) pattern proves that the structure of BCM does not change after 60 h. The results again matched well with the 2H hexagonal structure and were the same as those before the electrochemical test (see Figure 2). The HRTEM image in Figure 7b declares the [001] projection with a d spacing of 2.888 Å for (110) and 2.501 Å for (200). This *d* spacing matches with Pearson’s Crystal Data: 1900378. EDX mapping in Figure 7c demonstrates the distribution of the elements in hexagonal BCM. The results indicate that the elements (Ba, Co, Mn, and O) are homogeneously distributed in the material. Also, there is no segregation of any element shown in EDX mapping. This implies that elements are not segregated or do not become deficient after a long time of service. The after-TEM images show that 2H hexagonal BCM is stable during and after the electrochemical tests.

## 4 Conclusions

In summary, we report the application of a new hexagonal perovskite oxide in OER electrochemistry. Electrochemical OER activity and structure analysis of Ba_*x*_La_2–*x*_CoMnO_6−δ_ (*x* = 0, 0.5, 1, 1.5, 2) indicates that placing divalent ions at the site creates oxygen vacancies to keep the charge valence. BCM achieves 288 mV at 10 mA cm^–2^ and outstanding long-term stability in alkaline medium. The excellent OER activity of BCM is correlated with the oxygen vacancy formation in the structure and the low work function value in the electrocatalyst tested in this work. Post-OER characterization of BCM through HRTEM shows that the crystal structure remains, and no amorphization was observed. XPS analysis after ∼60 h of the stability experiment shows that oxidation states of Co and Mn do not change, while the oxygen vacancy concentration increases after the reaction suggesting that during the reaction, oxygen vacancies are generated and may also play a role in the reaction. These observations suggest that the A-site management strategy can be a promising strategy to boost the OER activity and structural stability of perovskite oxides.

## Supporting Information Available

XRD patterns for the Ba–Co–Mn–O system at different temperatures, Rietveld refined XRD patterns of synthesized catalysts, SEM images and EDS mappings, XPS survey spectra, XPS core-level spectra of Co 2p and Mn 2p, staircase CP, TOF, valence and Fermi spectra of catalysts, EPR spectra, OER performance under different pH conditions, Rietveld refinement and Goldschmidt tolerance factor results, BET results, comparison of overpotential, Tafel slope and TOF values, and work function calculations (PDF)
