# Supplementary material for: Facile Synthesis and Origin of Enhanced Electrochemical Oxygen Evolution Reaction Performance of 2H-Hexagonal Ba2CoMnO6−δ as a New Member in Double Perovskite Oxides
Source: ACS Omega. 2022 Nov 21;7(48):44147–55. doi: 10.1021/acsomega.2c05627 (PMC9730773; doi:10.1021/acsomega.2c05627)
Supplement: Supplementary file 1 — ao2c05627_si_001.pdf [file ao2c05627_si_001.pdf]

Facile synthesis and origin of enhanced electrochemical OER performance of 2H-Hexagonal Ba2CoMnO6-δ as a new member in double perovskite oxides

Tuncay Erdil[a], Ersu Lokcu[b], Ilker Yildiz[c], Can Okuyucu[a], Yunus Eren Kalay[a], Cigdem Toparli[a*]

[a] *Department of Metallurgical and Materials Engineering, Middle East Technical University, 06800, Ankara, Turkey*
[b] *Department of Metallurgical and Materials Engineering, Eskisehir Osmangazi University, 26040, Eskisehir, Turkey*
[c] *Central Laboratory, Middle East Technical University, 06800, Ankara, Turkey*

[*]Corresponding author.
E-mail address: ctoparli@ metu.edu.tr (C. Toparli)

List of Figures

Figure S1. (a) XRD patterns for Ba-Co-Mn-O system and zoom on diffraction peak zones placed between (b) 25-32° and (c) 52-57° 2θ degrees.

Figure S2. Rietveld refined XRD patterns of $Ba_xLa_{2-x}CoMnO_6$ (x = 0, 0.5, 1, 1.5, 2).

Figure S3. SEM images and EDS mapping of the (a) LCM, (b) BLCM-5, (c) BLCM, (d) BLCM-15, (e) BCM.

Figure S4. XPS survey spectra of $Ba_xLa_{2-x}CoMnO_{6-\delta}$ (x = 0, 0.5, 1, 1.5, 2)

Figure S5. XPS core level spectra of (a) Co2p and (b) Mn2p of LCM, BLCM-5, BLCM, BLCM-15, BCM

Figure S6. Staircase CP to determine the Tafel plot for $Ba_xLa_{2-x}CoMnO_{6-\delta}$ (x = 0, 0.5, 1, 1.5, 2)

Figure S7. TOF values calculated at η = 250, 300, 350, 400 and 450 mV

Figure S8. Valance and fermi spectra of $Ba_xLa_{2-x}CoMnO_6$ (x = 0, 0.5, 1, 1.5, 2)

Figure S9. Electro paramagnetic resonance (EPR) spectra of $Ba_xLa_{2-x}CoMnO_6$ (x = 0, 0.5, 1, 1.5, 2)

Figure S10. The OER performance of the catalysts under different pH conditions.

List of Tables

Table S1. Rietveld refinement analysis and Goldschmidt tolerance factor of double perovskite series of $Ba_xLa_{2-x}CoMnO_6$ (x = 0, 0.5, 1, 1.5, 2)

Table S2. BET Analysis

Table S3. Comparison of overpotential, tafel slope and TOF values with literature and this work.

Table S4. Corrected work function values.

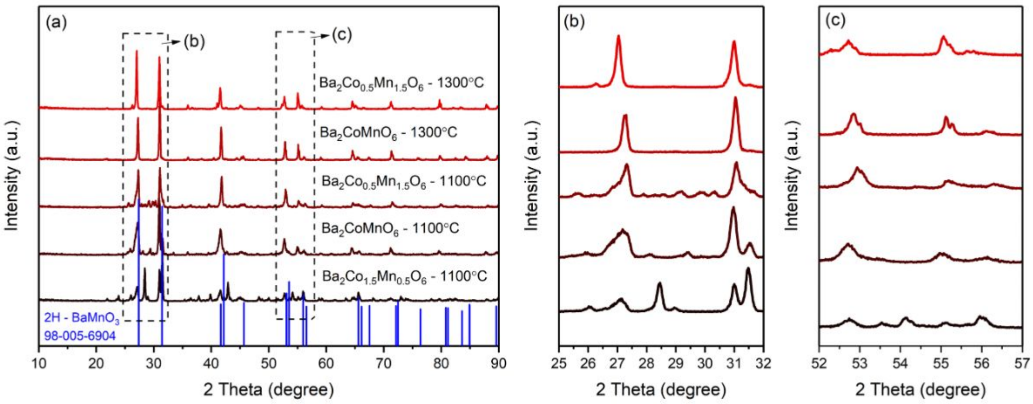

Figure S1. (a) XRD patterns for Ba-Co-Mn-O system and zoom on diffraction peak zones placed between (b) 25-32° and (c) 52-57° 2θ degrees.

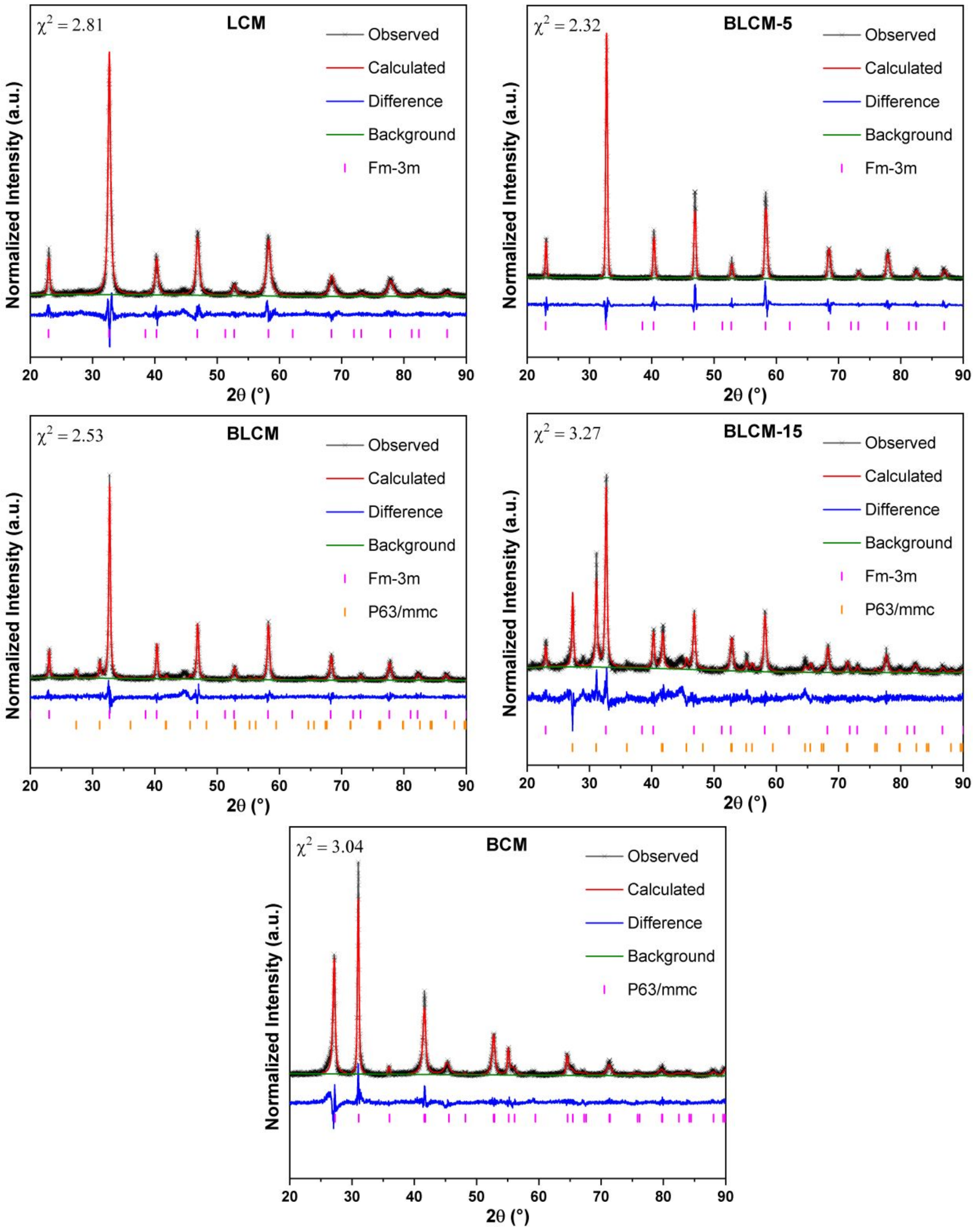

Figure S2. Rietveld refined XRD patterns of $Ba_xLa_{2-x}CoMnO_6$ (x = 0, 0.5, 1, 1.5, 2).

(a)

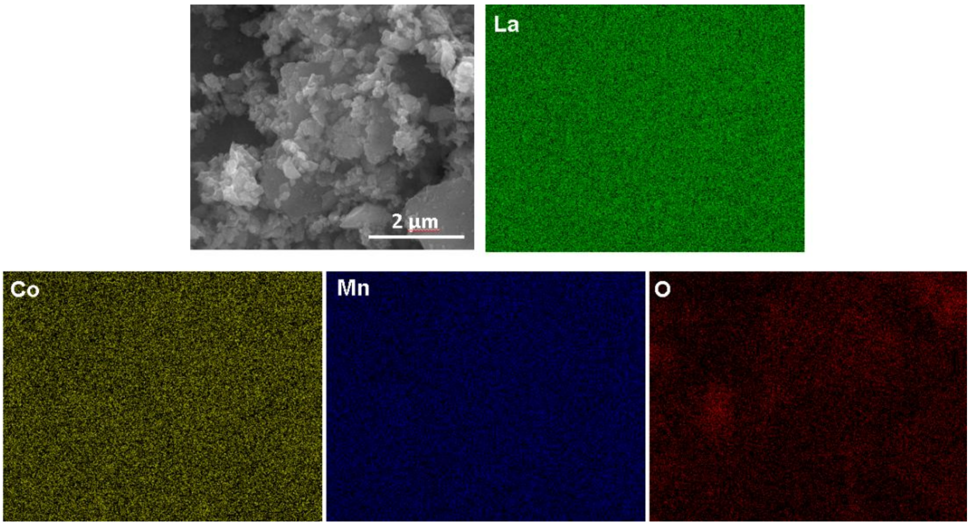

(b)

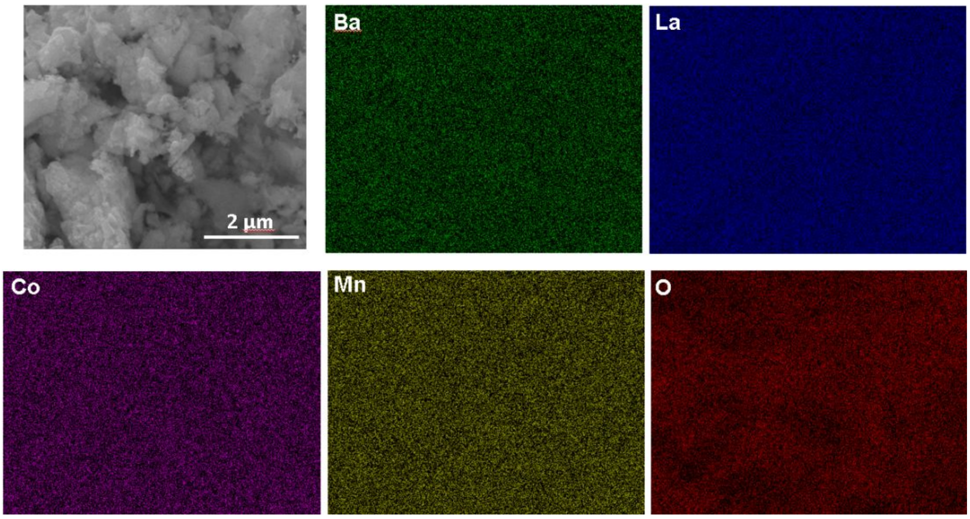

(c)

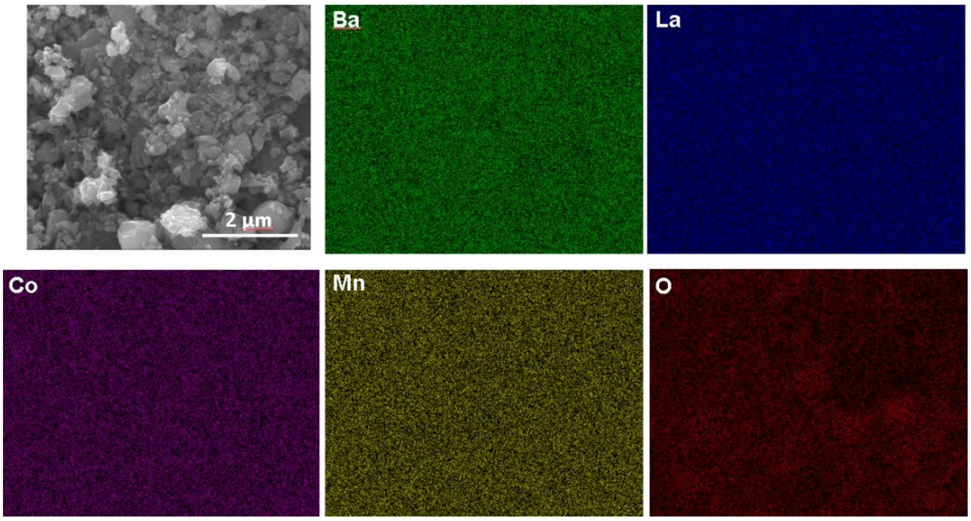

(d)

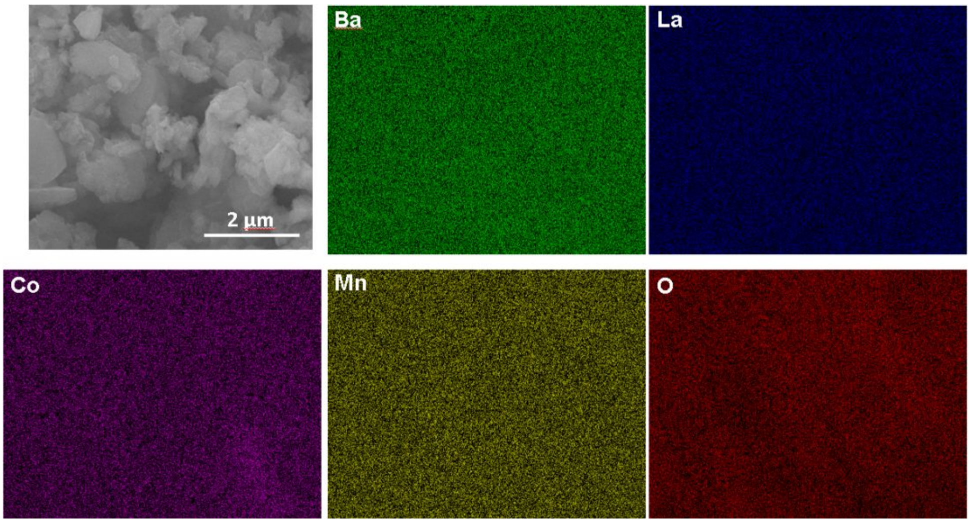

(e)

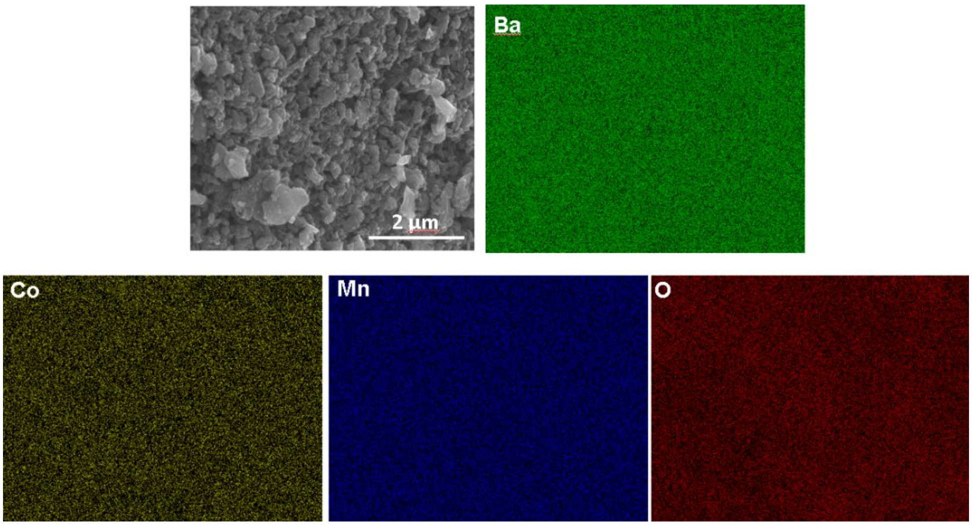

Figure S3. SEM images and EDS mapping of the (a) LCM, (b) BLCM-5, (c) BLCM, (d) BLCM-15, (e) BCM.

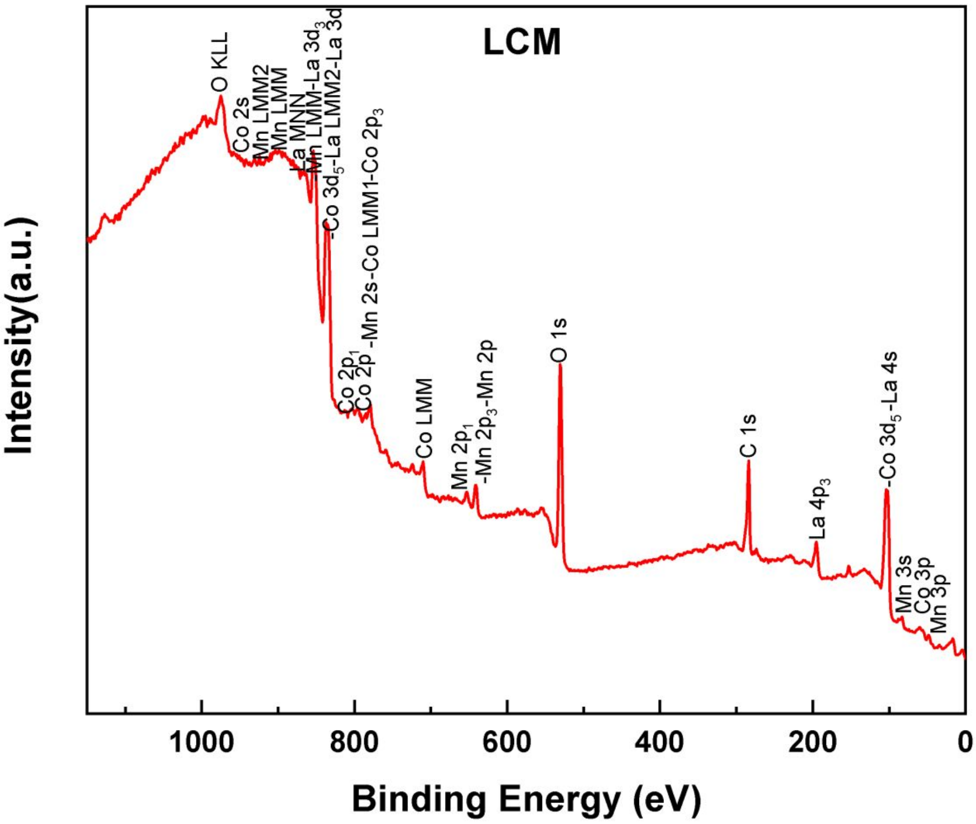
LCM
Intensity(a.u.)
Binding Energy (eV)
O KLL
O 1s
C 1s
La 4p3
Co LMM
1000
800
600
400
200
0

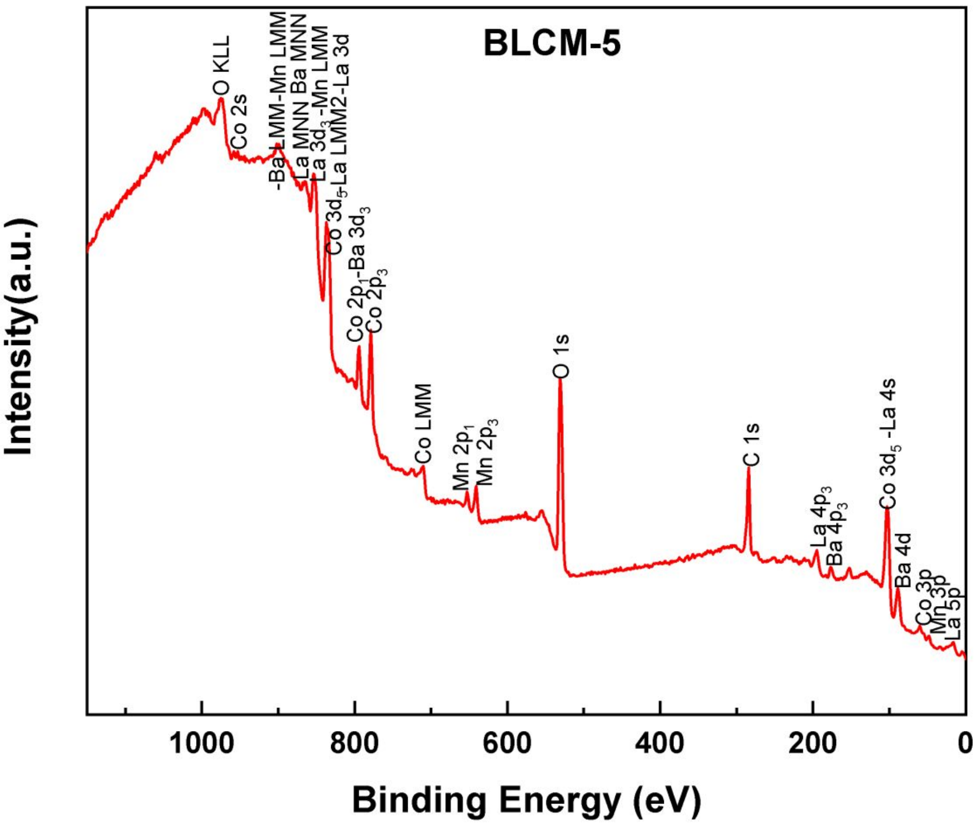
BLCM-5
Intensity(a.u.)
Binding Energy (eV)
O KLL
O 1s
C 1s
Co LMM
Ba 4d
1000
800
600
400
200
0

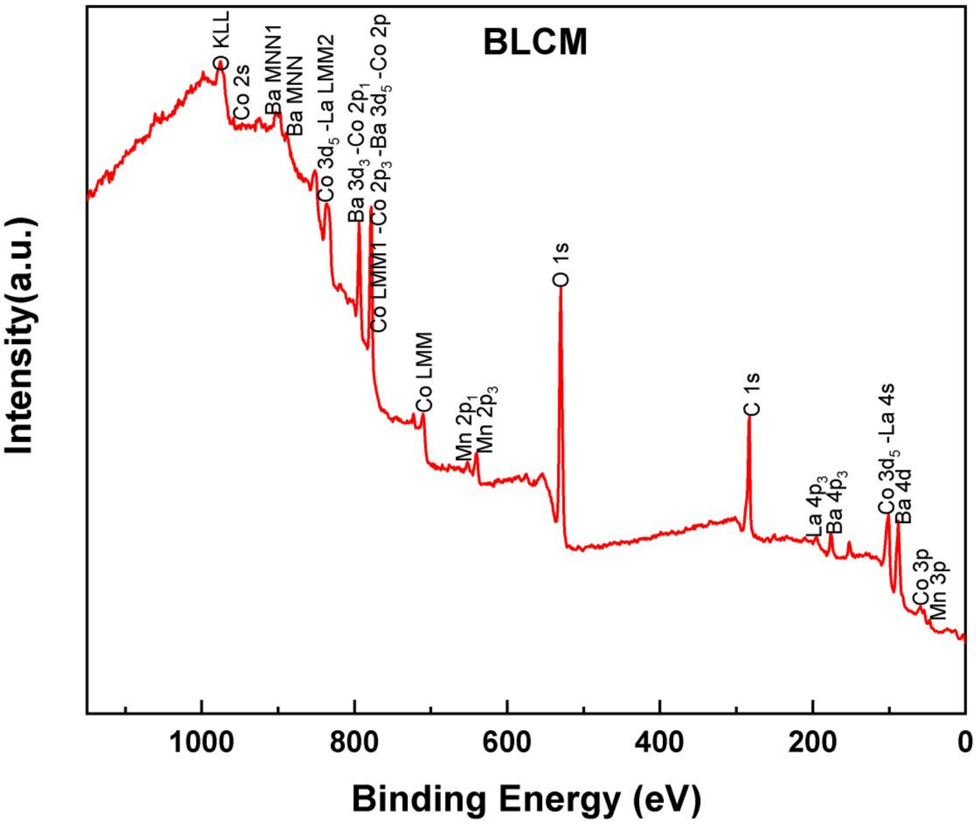
BLCM
Intensity(a.u.)
Binding Energy (eV)
1000
800
600
400
200
0
O KLL
Co 2s
Ba MNN1
Ba MNN
Co 3d5 -La LMM2
Ba 3d3 -Co 2p1
Co LMM1 -Co 2p3 -Ba 3d5 -Co 2p
Co LMM
Mn 2p1
Mn 2p3
O 1s
C 1s
La 4p3
Ba 4p3
Co 3d5 -La 4s
Ba 4d
Co 3p
Mn 3p

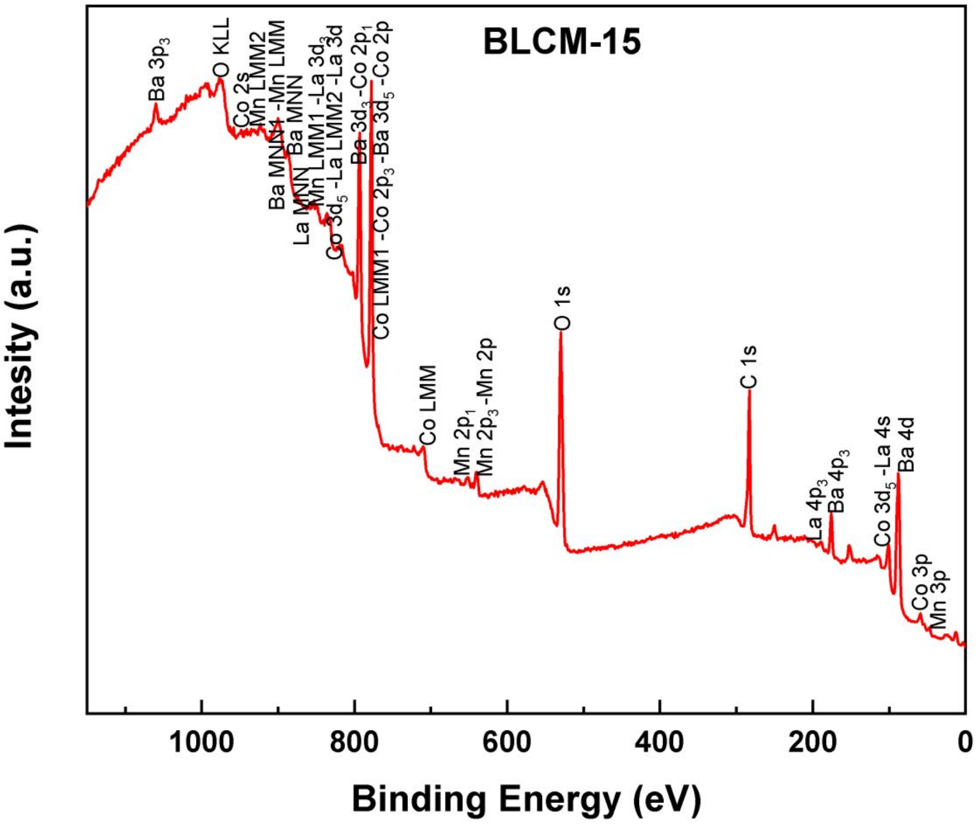
BLCM-15
Intesity (a.u.)
Binding Energy (eV)
1000
800
600
400
200
0
Ba 3p3
O KLL
Co 2s
Mn LMM2
Ba MNN1 -Mn LMM
Ba MNN
La MNN
Mn LMM1 -La 3d3
Co 3d5 -La LMM2 -La 3d
Ba 3d3 -Co 2p1
Co LMM1 -Co 2p3 -Ba 3d5 -Co 2p
Co LMM
Mn 2p1
Mn 2p3 -Mn 2p
O 1s
C 1s
La 4p3
Ba 4p3
Co 3d5 -La 4s
Ba 4d
Co 3p
Mn 3p

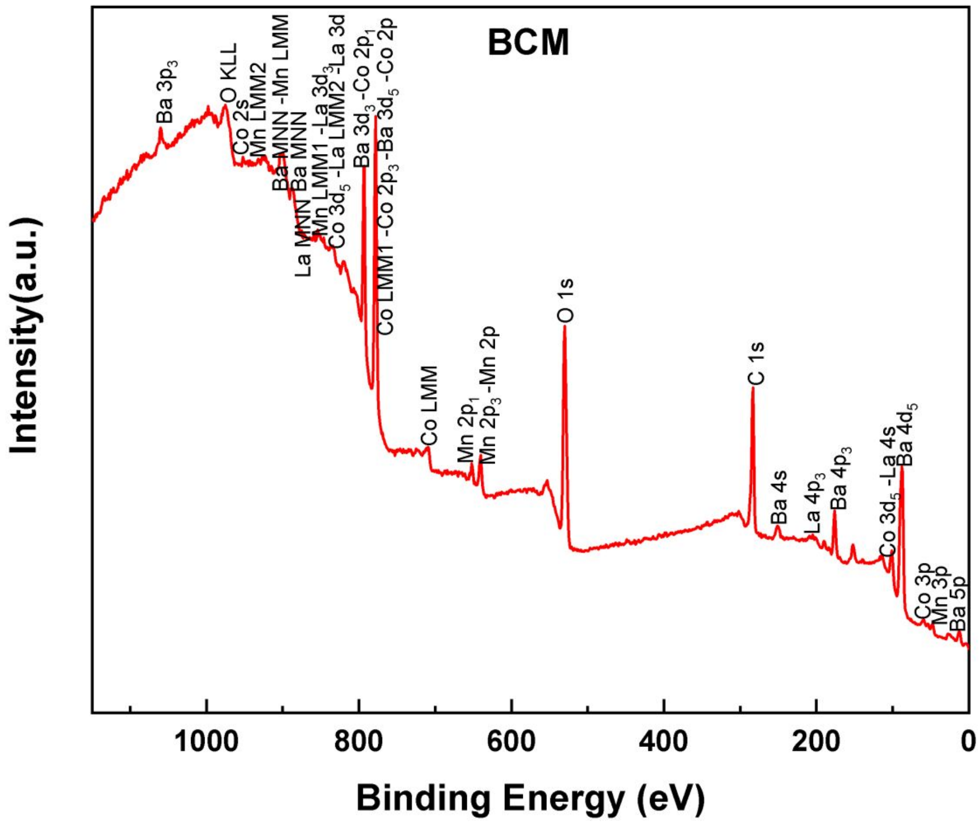

Figure S4. XPS survey spectra of $Ba_xLa_{2-x}CoMnO_{6-\delta}$ (x = 0, 0.5, 1, 1.5, 2)

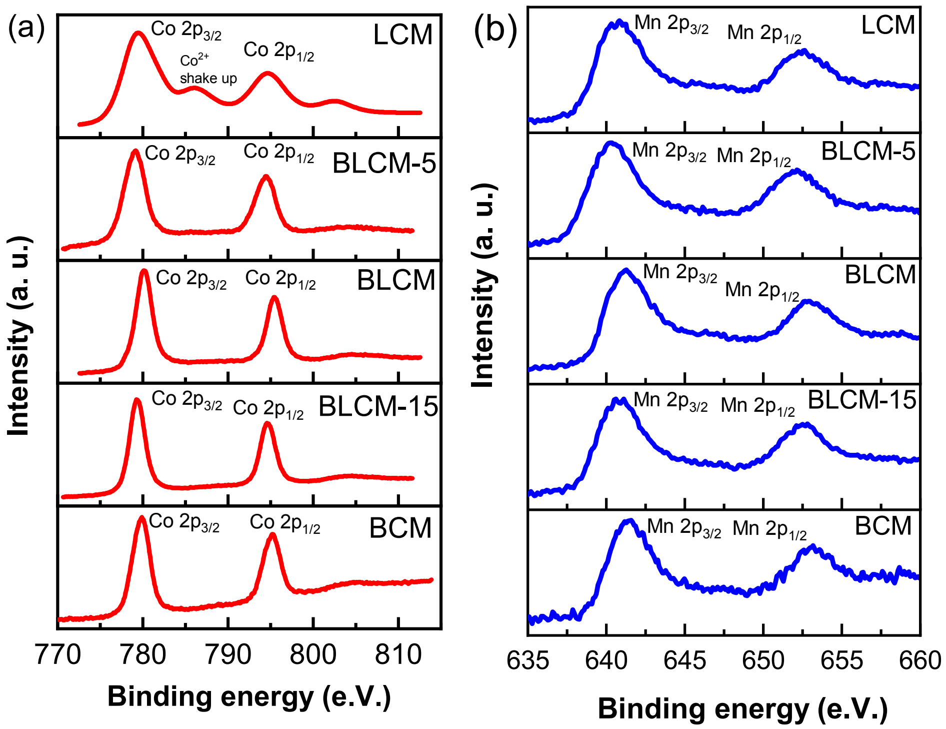

Figure S5. XPS core level spectra of (a) Co2p and (b) Mn2p of LCM, BLCM-5, BLCM, BLCM-15, BCM

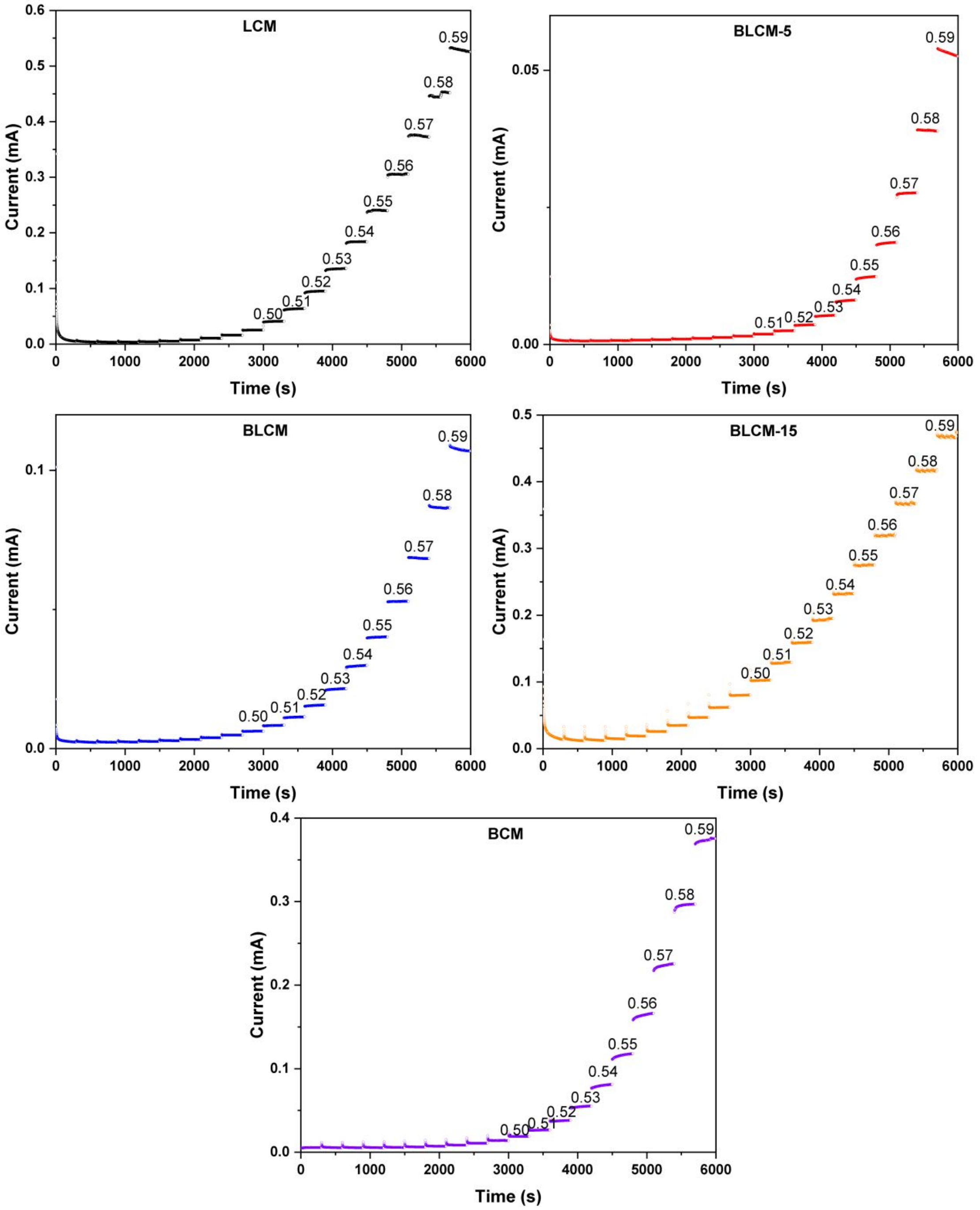

Figure S6. Staircase CP to determine the Tafel plot for $Ba_xLa_{2-x}CoMnO_{6-\delta}$ (x = 0, 0.5, 1, 1.5, 2)

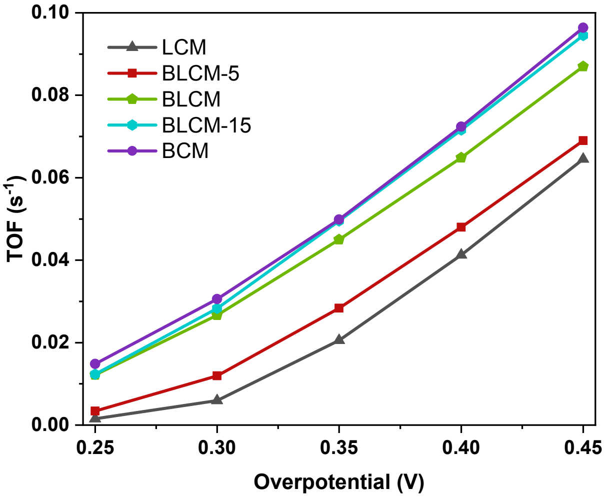

Figure S7. TOF values calculated at η = 250, 300, 350, 400 and 450 mV

(a) Au reference (stage)

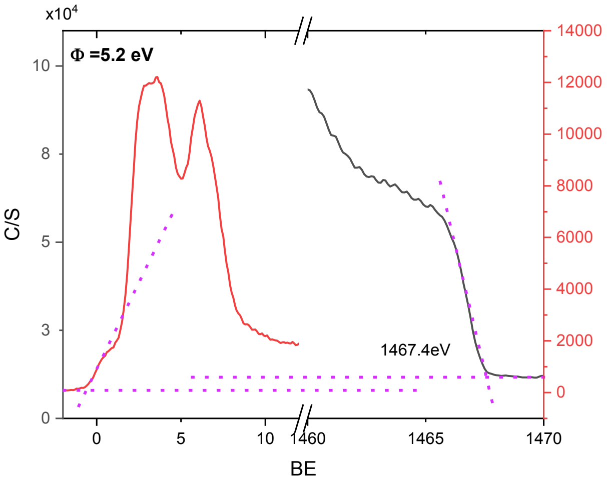

(b) La_2CoMnO_6

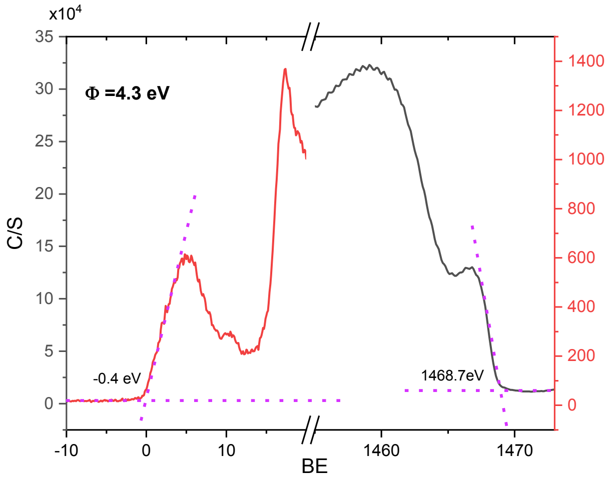

(c) $Ba_{0.5}La_{1.5}CoMnO_6$

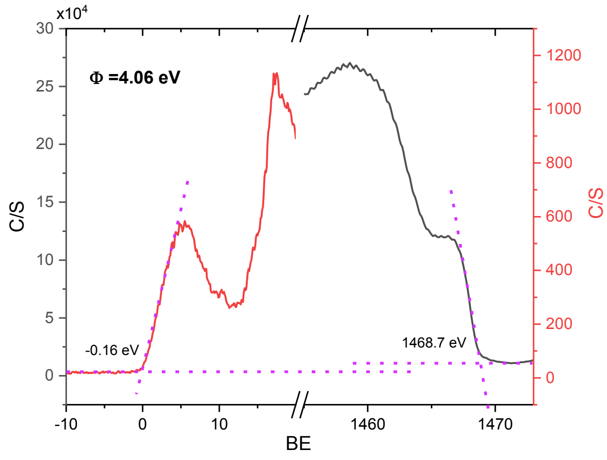

(d) $BaLaCoMnO_6$

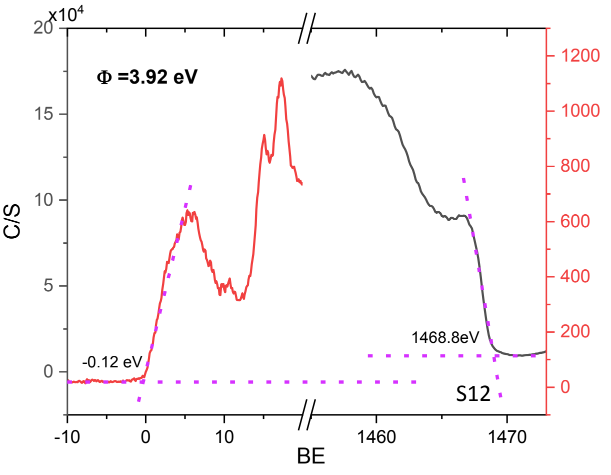

(e) $Ba_{1.5}La_{0.5}CoMnO_6$ **(f)** Ba_2CoMnO_6

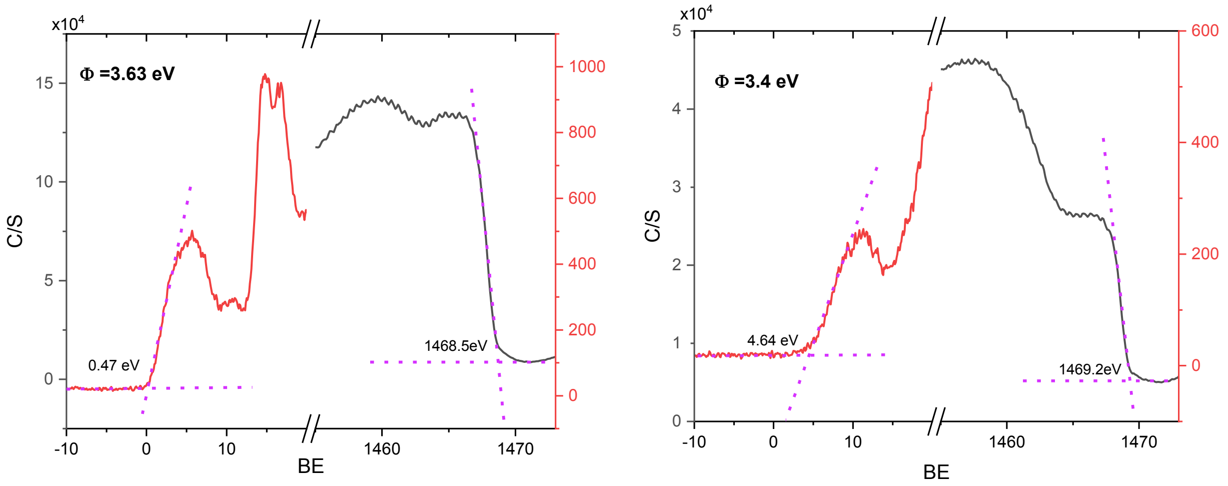

Figure S8. Valance and fermi spectra of (a) Au reference, (b) LCM, (c) BLCM-5, (d) BLCM, (e) BLCM-15, (f) BCM.

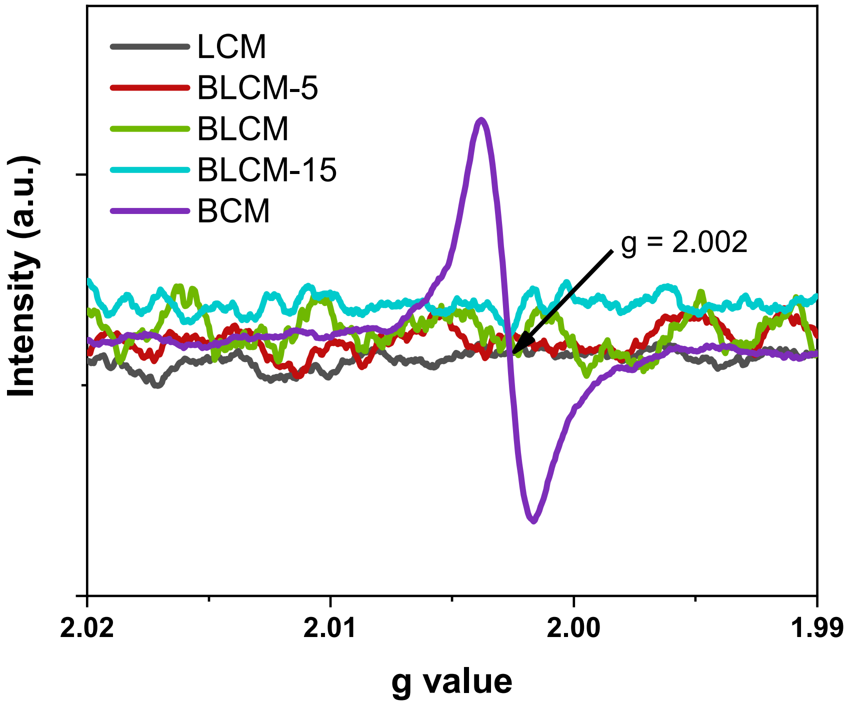

Figure S9. Electro paramagnetic resonance (EPR) spectra of $Ba_xLa_{2-x}CoMnO_6$ (x = 0, 0.5, 1, 1.5, 2)

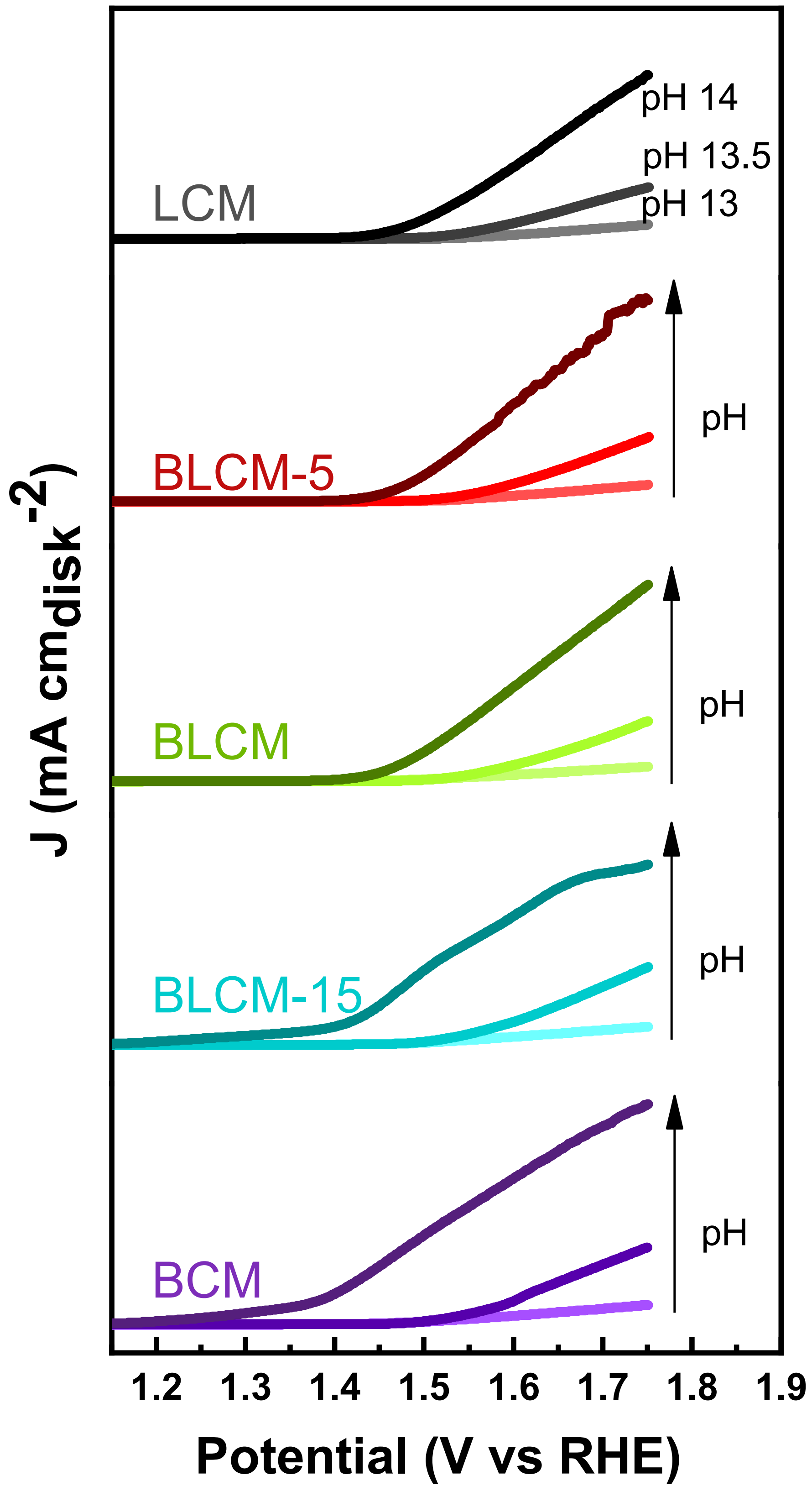

Figure S10. The OER performance of the catalysts under different pH conditions.

Table S1. Rietveld refinement analysis and Goldschmidt tolerance factor of double perovskite series of $Ba_xLa_{2-x}CoMnO_6$ (x = 0, 0.5, 1, 1.5, 2)

Catalyst	Lattice Parameter ($\AA$)			Space Group	Volume ($\AA^3$)	Tolerance Factor
	a	b	c			
LCM	7.76	7.76	7.76	$Fm\bar{3}m$	466.620	0.991
BLCM-5	7.77	7.77	7.77	$Fm\bar{3}m$	469.097	0.997
BLCM	7.79	7.79	7.79	$Fm\bar{3}m$(90wt%)	472.365	1.023
	5.78	5.78	4.35	$P6_3/mmc$ (10wt%)	125.706	
BLCM-15	7.79	7.79	7.79	$Fm\bar{3}m$(59wt%)	471.99	1.045
	5.78	5.78	4.35	$P6_3/mmc$(41wt%)	125.810	
BCM	5.77	5.77	4.37	$P6_3/mmc$	126.362	1.063

Sample	Multipoint BET				Single Point BET	
	P/P_0	Volume(cc/g)	Slope	Area(m^2/g)	P/P_0	Area(m^2/g)
LCM	0.06112	2.5671	173.9	18.90	0.31144	18.78
	0.08765	2.9831				
	0.11267	3.3101				
	0.16424	3.9766				
	0.21310	4.6115				
BLCM-5	0.06059	2.2213	239.8	13.99	0.31222	14.82
	0.08756	2.5237				
	0.11270	2.7681				
	0.16231	3.2281				
	0.21225	3.5966				
BLCM-15	0.11260	3.7133	132.2	23.96	0.31212	19.90
	0.16231	4.3159				
	0.21161	5.2742				
	0.26221	6.0080				
	0.31212	6.6472				
BLCM	0.08743	3.1955	156.2	20.82	0.31207	18.12
	0.11256	3.5191				
	0.16226	4.0865				
	0.21154	4.9035				
	0.26209	5.5099				
BCM	0.06044	2.7515	160.3	20.49	0.31119	19.41
	0.08751	3.1486				
	0.11227	3.6338				
	0.16214	4.3677				

Table S2. BET Analysis

Table S3. Comparison of overpotential, tafel slope and TOF values with literature and this work.

Electrocatalyst	Overpotential (mV)	Tafel Slope	TOF (s^{-1}) @300-400 mV n
BCM	288	50	0.031-0.072
BLCM-15	295	85	0.028-0.071
BLCM	300	73	0.027-0.065
BLCM-5	346	57	0.012-0.048
LCM	365	56	0.006-0.041
$SrNb_{0.1}Co_{0.7}Fe_{0.2}O_{3-\delta}$[1]	400	38.3	0.024
$La(CrMnFeCo_2Ni)O_3$[2]	325	51.2	0.027
$(FeCoNiCrMn)_3O_4$[3]	288	60	0.159
α-$Co_4Fe(OH)_x$[4]	295	52	0.027 @300 mV
$MnCoFeO_4$[5]	480	-	0.011 @300 mV
$NdBaMn_2O_{5.5}$[6]	430	75	
$Ba_2CoMo_{0.5}Nb_{0.5}O_{6-\delta}$[7]	445	77	
$Sr_2Fe_{0.8}Co_{0.2}Mo_{0.65}Ni_{0.35}O_{6-\delta}$[8]	310	56	

The TOF value is calculated by the following equation: $TOF = \frac{JA}{4nF}$

j is the measured current density (mA cm^{-2}), A is the surface area of the GC electrode (0.07068 cm^2), n is the moles of CoMn deposited on the electrode, F is the Faraday constant (96485 C mol^{-1}).

Table S4. Corrected work function values.

	Cut off (BE/eV)	Fermi Level (BE/ev)	φ, Work function (eV)	φ, Work function (eV)(corrected with Au coating) (eV)
Au coating on LCM	1468.5	-0.79	4.89	*5.2
La_2CoMnO_6(LCM)	1468.7	-0.40	4.37	4.68
Au coating on BLCM-5	1468.7	-0.73	4.63	
$Ba_{0.5}La_{1.5}CoMnO_6$(BLCM-5)	1468.7	-0.16	4.06	4.63
Au coating on BLCM	1468.5	-0.74	4.84	
$BaLaCoMnO_6$(BLCM)	1468.8	-0.12	3.92	3.56
Au coating on BLCM-15	1468.5	-0.74	4.84	
$Ba_{1.5}La_{0.5}CoMnO_6$(BLCM-15)	1468.5	0.47	3.63	3.27
Au coating on BCM	1468.3	-0.74	5.04	
Ba_2CoMnO_6(BCM)	1469.2	4.64	3.40	3.24

References

(1) Liu, Q.; Zhu, Y.; He, Z.; Jin, S.; Chen, Y. A Facile Top-down Approach for Constructing Perovskite Oxide Nanostructure with Abundant Oxygen Defects as Highly Efficient Water Oxidation Electrocatalyst. *Int J Hydrogen Energy* **2020**, *45* (43), 22808–22816. https://doi.org/10.1016/j.ijhydene.2020.06.137.

(2) Nguyen, T. X.; Liao, Y.; Lin, C.; Su, Y.; Ting, J. Advanced High Entropy Perovskite Oxide Electrocatalyst for Oxygen Evolution Reaction. *Adv Funct Mater* **2021**, *31* (27), 2101632. https://doi.org/10.1002/adfm.202101632.

(3) Duan, C.; Li, X.; Wang, D.; Wang, Z.; Sun, H.; Zheng, R.; Liu, Y. Nanosized High Entropy Spinel Oxide $(FeCoNiCrMn)_3O_4$ as a Highly Active and Ultra-Stable Electrocatalyst for the Oxygen Evolution Reaction. *Sustain Energy Fuels* **2022**, *6* (6), 1479–1488. https://doi.org/10.1039/D1SE02038B.

(4) Jin, H.; Mao, S.; Zhan, G.; Xu, F.; Bao, X.; Wang, Y. Fe Incorporated α-$Co(OH)_2$ Nanosheets with Remarkably Improved Activity towards the Oxygen Evolution Reaction. *J Mater Chem A Mater* **2017**, *5* (3), 1078–1084. https://doi.org/10.1039/C6TA09959A.

(5) Zhan, Y.; Xu, C.; Lu, M.; Liu, Z.; Lee, J. Y. Mn and Co Co-Substituted Fe_3O_4 Nanoparticles on Nitrogen-Doped Reduced Graphene Oxide for Oxygen Electrocatalysis in Alkaline Solution. *J. Mater. Chem. A* **2014**, *2* (38), 16217–16223. https://doi.org/10.1039/C4TA03472D.

(6) Wang, J.; Gao, Y.; Chen, D.; Liu, J.; Zhang, Z.; Shao, Z.; Ciucci, F. Water Splitting with an Enhanced Bifunctional Double Perovskite. *ACS Catal* **2018**, *8* (1), 364–371. https://doi.org/10.1021/acscatal.7b02650.

(7) Sun, H.; Chen, G.; Sunarso, J.; Dai, J.; Zhou, W.; Shao, Z. Molybdenum and Niobium Codoped B-Site-Ordered Double Perovskite Catalyst for Efficient Oxygen Evolution Reaction. *ACS Appl Mater Interfaces* **2018**, *10* (20), 16939–16942. https://doi.org/10.1021/acsami.8b03702.

(8) Sun, H.; Xu, X.; Hu, Z.; Tjeng, L. H.; Zhao, J.; Zhang, Q.; Lin, H.-J.; Chen, C.-T.; Chan, T.-S.; Zhou, W.; Shao, Z. Boosting the Oxygen Evolution Reaction Activity of a Perovskite through Introducing Multi-Element Synergy and Building an Ordered Structure. *J Mater Chem A Mater* **2019**, *7* (16), 9924–9932. https://doi.org/10.1039/C9TA01404G.
